# ceRNA regulatory network and immune-neurodegenerative mechanisms of peripheral CD4+ T cells in parkinson’s disease

**DOI:** 10.1371/journal.pone.0337808

**Published:** 2025-12-02

**Authors:** Lijun Guo, Qiong Li, Jingyi Li, Feng Yang

**Affiliations:** 1 Department of Neurology, Affiliated Hospital Of Xiangnan University, Chenzhou, China; 2 Department of Intensive Care Medicine, Chenzhou NO.1 People’s Hospital, Chenzhou, China; 3 Institute of Pharmacology, Xuzhou Medical University, Xuzhou China; Ondokuz Mayis University Faculty of Medicine: Ondokuz Mayis Universitesi Tip Fakultesi, TÜRKIYE

## Abstract

Parkinson’s disease (PD) is a neurodegenerative disorder characterized by dopaminergic neuron loss and neuroinflammation, with emerging roles of peripheral immune dysregulation in disease progression. This study aimed to investigate the regulatory network of CD4 + T cells in PD through multi-omics integrative analysis. Transcriptomic and miRNA datasets from peripheral blood mononuclear cells (PBMCs) of 20 PD patients and 17 healthy controls were analyzed (GSE22491, GSE100054, GSE16658). Differential expression analysis identified 287 mRNAs and 73 miRNAs (|log₂(fold change)| ≥ 0.5, false discovery rate <0.05), with 25 immune-related mRNAs selected for further analysis. Machine learning (LASSO regression and SVM-RFE) prioritized seven hub genes, including *CD4* and *SEMA6A*. A competing endogenous RNA (ceRNA) network was constructed, comprising 38 lncRNAs, three miRNAs (miR-155-5p, miR-27a-3p, miR-27b-3p), and their target mRNAs *CD4* and *SEMA6A*. Four lncRNAs (including *XIST, NORAD*, and *INE1*) were identified to functionally regulate *CD4*. Immune cell infiltration analysis revealed increased proportions of naïve CD4 + T cells and activated dendritic cells in PD patients. *CD4* expression positively correlated with γδ T cells (r = 0.48, p = 0.032) and activated NK cells (r = 0.45, p = 0.048). Gene set enrichment analysis associated *CD4* with neurodegenerative pathways (e.g., Parkinson’s disease: normalized enrichment score = 1.57, *p* = 0.002) and oxidative phosphorylation (normalized enrichment score = 1.89, *p* = 7.4 × 10 ⁻ ⁶). These findings highlight a peripheral CD4 + T cell-centric ceRNA network that modulates immune-metabolic crosstalk and neuroinflammation in PD. This study provides novel insights into immune-driven neurodegeneration and suggests potential therapeutic targets for PD through metabolic-immune reprogramming.

## Introduction

Parkinson’s disease (PD) is a chronic neurodegenerative disorder marked by the progressive loss of dopaminergic neurons in the substantia nigra and the abnormal accumulation of α-synuclein within neurons, forming Lewy bodies [1]. Clinically, PD is primarily manifested by motor symptoms, including resting tremor, rigidity, bradykinesia, and postural instability, along with non-motor symptoms such as cognitive impairment, sleep disturbances, and autonomic dysfunction [2]. Although the exact etiology of PD remains unclear, complex interactions between genetic susceptibility (e.g., mutations in LRRK2 and SNCA), environmental toxin exposure, age-related oxidative stress, and immune system abnormalities have been implicated in disease progression [3,4]. Traditionally, PD pathology was considered to be confined to the central nervous system; however, recent studies have revealed the critical role of the peripheral immune system in neuroinflammation and neuronal damage, particularly the central effector cells of adaptive immunity—CD4 + T cells [5].

CD4 + T cells regulate immune homeostasis through a dynamic balance between their functional subsets (Th1, Th2, Th17, and regulatory T cells/Tregs). Disruption of this balance in PD may drive neurodegeneration: IFN-γ and TNF-α secreted by Th1 cells can exacerbate neuroinflammation by activating microglia and directly damaging dopaminergic neurons [6]; IL-17A produced by Th17 cells disrupts the blood-brain barrier, promoting the infiltration of peripheral immune cells [7]; and Treg dysfunction results in reduced immune suppression, further amplifying the inflammatory response [8]. Notably, CD4 + T cells may recognize α-synuclein epitopes via molecular mimicry, triggering autoimmune responses against dopaminergic neurons [9], a process supported by the detection of α-synuclein-specific T cell receptor (TCR) clones in the serum and cerebrospinal fluid of PD patients [10].

Recent studies have extended the theoretical framework of the “gut-brain axis” in PD pathology. Over 80% of PD patients experience gastrointestinal dysfunction (e.g., constipation), and as the largest peripheral immune organ in the body, the gut’s abnormal activation of CD4 + T cells may influence the central nervous system via the vagus nerve or systemic inflammation [11]. The gut microbiome modulates CD4 + T cell differentiation through mechanisms such as the metabolism of short-chain fatty acids (SCFAs), with a reduction in Prevotella abundance being significantly associated with Th17/Treg imbalance [12]. This gut-derived immune dysregulation may occur before the onset of motor symptoms, suggesting its potential value in early diagnosis and intervention of PD [13].

Based on the aforementioned background, CD4 + T cells have emerged as a key target for immunotherapy in PD. However, the molecular regulatory networks of CD4 + T cells in peripheral immunity, inter-organ interactions, and disease stage-specific dynamic changes remain to be systematically elucidated. This study integrates multi-omics data to identify key peripheral immune genes in PD, construct a *CD4*-related ceRNA regulatory network, and explore the relationship between *CD4* expression and immune cell infiltration. The findings will deepen our understanding of the peripheral immune mechanisms in PD and provide new strategies for immune-metabolic reprogramming-based precision therapy.

## Materials and methods

### Data collection and study participants

The mRNA and miRNA datasets were obtained from the National Center for Biotechnology Information’s GEO database (https://www.ncbi.nlm.nih.gov/geo/), where the keywords searched were “Parkinson’s disease” and “peripheral blood mononuclear cell”. The species was identified as Homo sapiens. After screening, three datasets were included, including two mRNA (GSE22491 and GSE100054) datasets and one miRNA (GSE16658) dataset. The key characteristics of the study participants from each dataset, as reported in the original publications, are summarized below to provide essential clinical context. GSE100054 (Platform GPL23126) included whole transcriptome data from PBMCs of 10 PD patients and 9 healthy controls [14]. PD patients were at mild to moderate disease stages (Hoehn & Yahr stages I-III) and showed reduced cardiac uptake of meta-iodobenzylguanidine, supporting the diagnosis. Patients with dementia with Lewy bodies or known familial PD were excluded. The average age was 65.3 years for patients and 62.1 years for controls. GSE22491 (Platform GPL6480) contained whole transcriptome data from PBMCs of 10 PD patients and 8 HCs [15]. The PD cohort consisted exclusively of patients carrying the LRRK2 G2019S mutation. Clinical status was defined using Gelb’s diagnostic criteria, ranging from “asymptomatic carrier” to “probable PD”. The mean age at sampling was 68.8 ± 11.0 years for the PD group and 68.6 ± 12.0 years for controls. GSE16658 (Platform 7722) provided miRNA transcriptome data from PBMCs of 19 PD patients and 13 HCs [16]. The patients covered a broad spectrum of disease severity, from early to advanced stages (Hoehn & Yahr stages 1–5). The average age at examination was 65.1 ± 4.4 years for patients and 64.4 ± 5.9 years for controls. Next, we obtained a curated list of 2483 immune-related genes (updated 2023) from IMMPORT (https://immport.org)

### Differential mRNA and miRNA expression analyses

The “edgeR” package of R software was used to merge, remove batch effect and normalize the two mRNA datasets. The differential expression mRNAs (DEmRNAs) of PD patients and healthy controls were identified by “limma” package of R software, mRNAs that met the criteria of |log_2_FC| ≥ 0.5 and FDR < 0.05 were considered as DEmRNA. Through the GEO2R online tool, we obtained the differential expression miRNAs (DEmiRNAs), miRNAs that met the criteria of |log_2_FC| ≥ 0.5 and FDR < 0.05 were considered as DEmiRNA.

### Immunorelated DEmRNAs screening

By intersecting DEmRNAs with immune-related genes, we identified immune-related DEmRNAs.

### Machine learning methods screen hub immune genes

The immune-related DEmRNAs were screened by lasso regression and support vector machine, and the key immune-related mRNAs were obtained by intersection

### Building the ceRNA network

The miRNAs of immune-related mRNAs were searched in the mirtarbase database (https://mirtarbase.cuhk.edu.cn/), and all the miRNAs were confirmed by literature. The obtained miRNAs intersected with the DEmiRNAs. Finally, lncRNA of miRNA was searched in the StarBase database (https://rnasysu.com/encori/), and ceRNA network was established using cytoscape software.

### Immune infiltration analysis by CIBERSORT and the correlation between key genes and infiltrating immune cells

CIBERSORT was used to analyze the proportion of various immune cells in the PBMC of PD patients and healthy controls according to the principle of linear support vector regression. We then studied the relationship between mRNA in the ceRNA network and the infiltration rate of immune cells, and calculated pearson’s correlation coefficient and P-value (p value <0.05 was the threshold of significance). Finally, we used the R package “ggplot2” to draw a lollipop map of the association of *CD4* genes with different immune cells.

## Results

### Batch effect correction of the data

**Fig 1:**
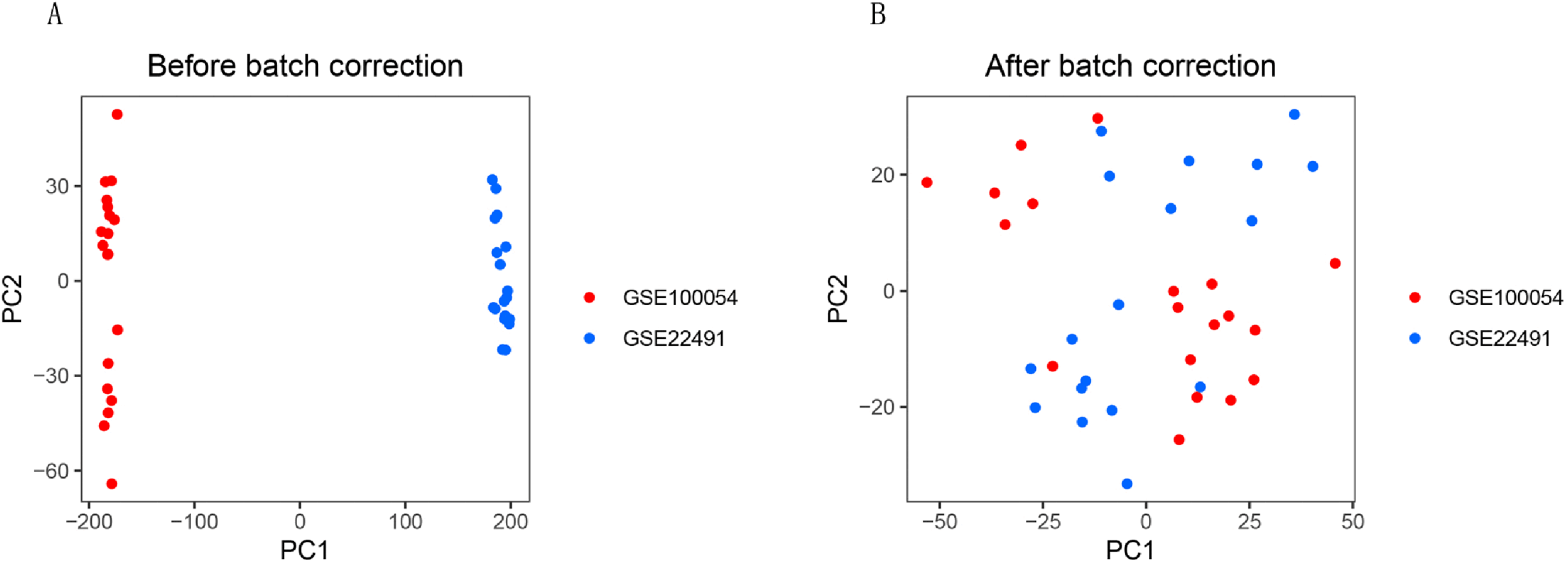
Quality assessment of GSE10054 and GSE22491 before and after batch effect correction. **(A)** Principal component analysis (PCA) before batch effect correction. **(B)** PCA plot after batch effect correction.

The two data sets were combined and batch corrected to create a PD data set. The dataset included 20 Parkinson’s patients and 17 normal control samples. We evaluated the data quality before and after batch effect correction, and generated a PCA map (Fig 1, mRNA expression profile before and after batch effect correction). The results showed that the distribution of gene expression was uneven in the two data sets before correction, and the differences in principal components were significant (Fig 1A). In contrast, after correction, the expression of genes in the two datasets was evenly distributed, with little difference in principal components (Fig 1B), indicating that the batch effect was effectively reduced, which could improve subsequent data analysis.

### Screening for differentially expressed mRNAs and miRNAs

**Fig 2:**
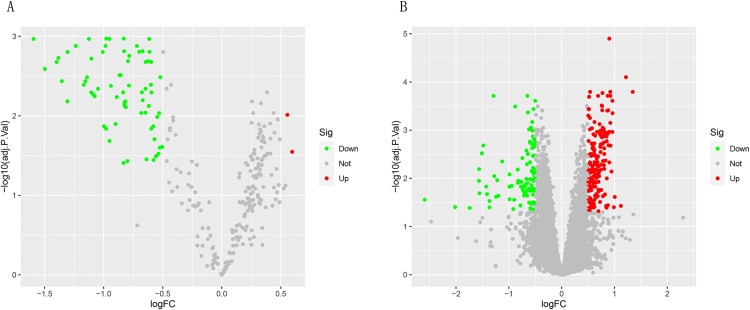
Analysis of differentially expressed genes. **(A)** Volcanic map showing the DEmiRNAs between PD and healthy controls. **(B)** Volcanic map showing the DEmRNAs between PD and healthy controls.

We analyzed the differences between the combined mRNA expression profile data and the miRNA data set, and used the volcano map (Fig 2) to show the stage screening results of differentially expressed genes. A total of 287 differentially expressed mRNAs were screened, including 181 up-regulated mRNAs and 106 down-regulated mRNAs. A total of 73 miRNAs were differentially expressed, including 2 up-regulated miRNAs and 71 down-regulated miRNAs.

### Identification of immune-associated DEmRNAs

**Fig 3:**
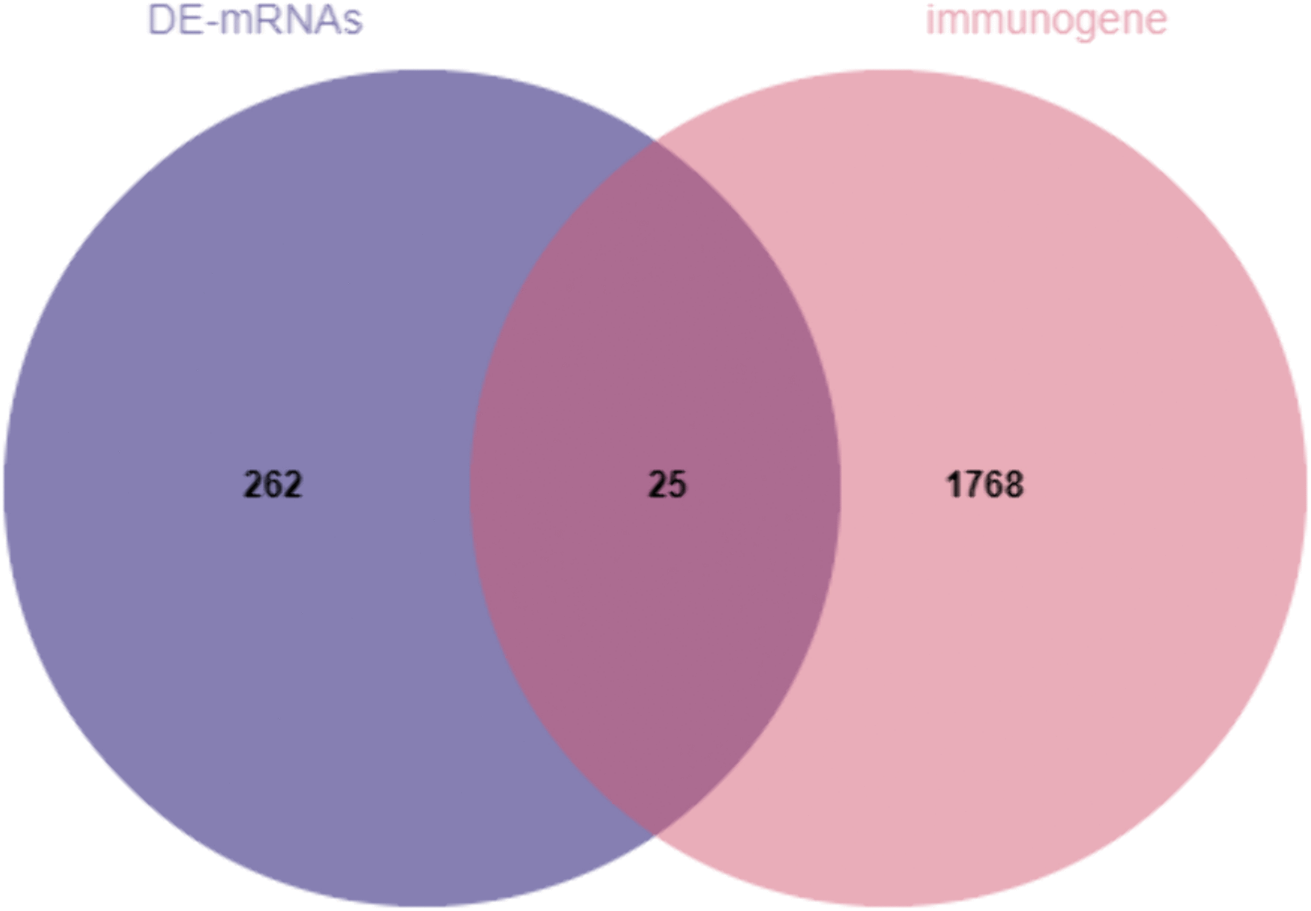
Venn diagram of DEmRNAs and immune-related genes.

The intersection of DEmRNAs and immune-related genes was analyzed and a Venn diagram was made (Fig 3). 25 immune-related DEmRNAs were obtained. Among them, the 12 down-regulated are *LTF, DEFA4, ELANE, PGLYRP1, CAMP, PI3, SLPI, AZU1, IL7, IGKV1D-8, TMSB15A, CD3G, EBI3*, and the 13 up-regulated are: *SRC, GDNF, SEMA6A, UCN2, GIPR, PLXND1, CRHR2, AVP, SEMA6B, CCR4, CD4, CLEC4M, EBI3*.

### Machine learning methods screen key genes

**Fig 4:**
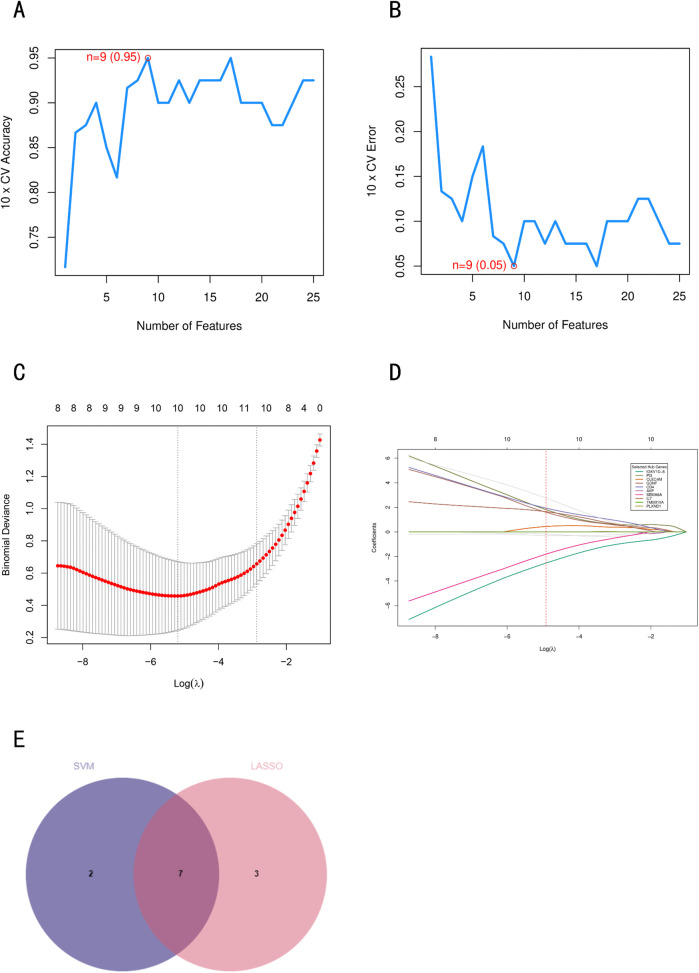
Key gene identification based on two machine learning algorithms. The LASSO logistic regression model was applied to screen the hub gene, and the partial likelihood bias of 10-fold cross-validation was used to calculate the optimal λ **(C, D)**. Accuracy **(A)** and error **(B)** of feature selection in SVM-RFE algorithm for hub gene selection. **(E)** Venn diagrams of hub genes screened by two machine learning algorithms.

We employed two machine learning algorithms to refine candidate hub genes from the 25 immune-related differentially expressed genes. LASSO regression identified 10 candidate mRNAs at optimal lambda values. Subsequently, SVM-RFE analysis selected nine mRNAs (Fig 4), with seven genes common to both methods: *PI3, TMSB15A, CLEC4M, CD4, SEMA6A, PLXND1*, and *AVP.*

The optimal feature number (n = 9) for SVM-RFE was determined by peak performance metrics: maximal 10-fold cross-validation accuracy (0.95) and minimal error rate (0.05) (Fig 4A, 4B). Comprehensive evaluation demonstrated superior F1-score (0.995) and recall (0.99) for the 9-gene set compared to adjacent feature sets (n = 8: F1 = 0.99; n = 10: F1 = 0.985), indicating enhanced robustness for PD classification (Table 1). The inclusion of AVP (arginine vasopressin) is biologically significant, as this neurohypophysial hormone participates in neuro-immune-endocrine regulation and may contribute to PD pathogenesis through neuroinflammatory pathways and stress response mechanisms.

**Table 1:** Performance metrics of SVM-RFE feature sets.

Number of Features	Gene List	AUC	Accuracy	Precision	Recall	F1-score
8	*PI3; TMSB15A; SEMA6A; CD4; CLEC4M; EBI3; PLXND1; GIPR*	1	0.989	1	0.98	0.99
9	*PI3; TMSB15A; SEMA6A; CD4; CLEC4M; EBI3; PLXND1; GIPR; AVP*	1	0.995	1	0.99	0.995
10	*PI3; TMSB15A; SEMA6A; CD4; CLEC4M; EBI3; PLXND1; GIPR; AVP; CAMP*	1	0.984	1	0.97	0.985

### Construction of the ceRNA network

**Fig 5:**
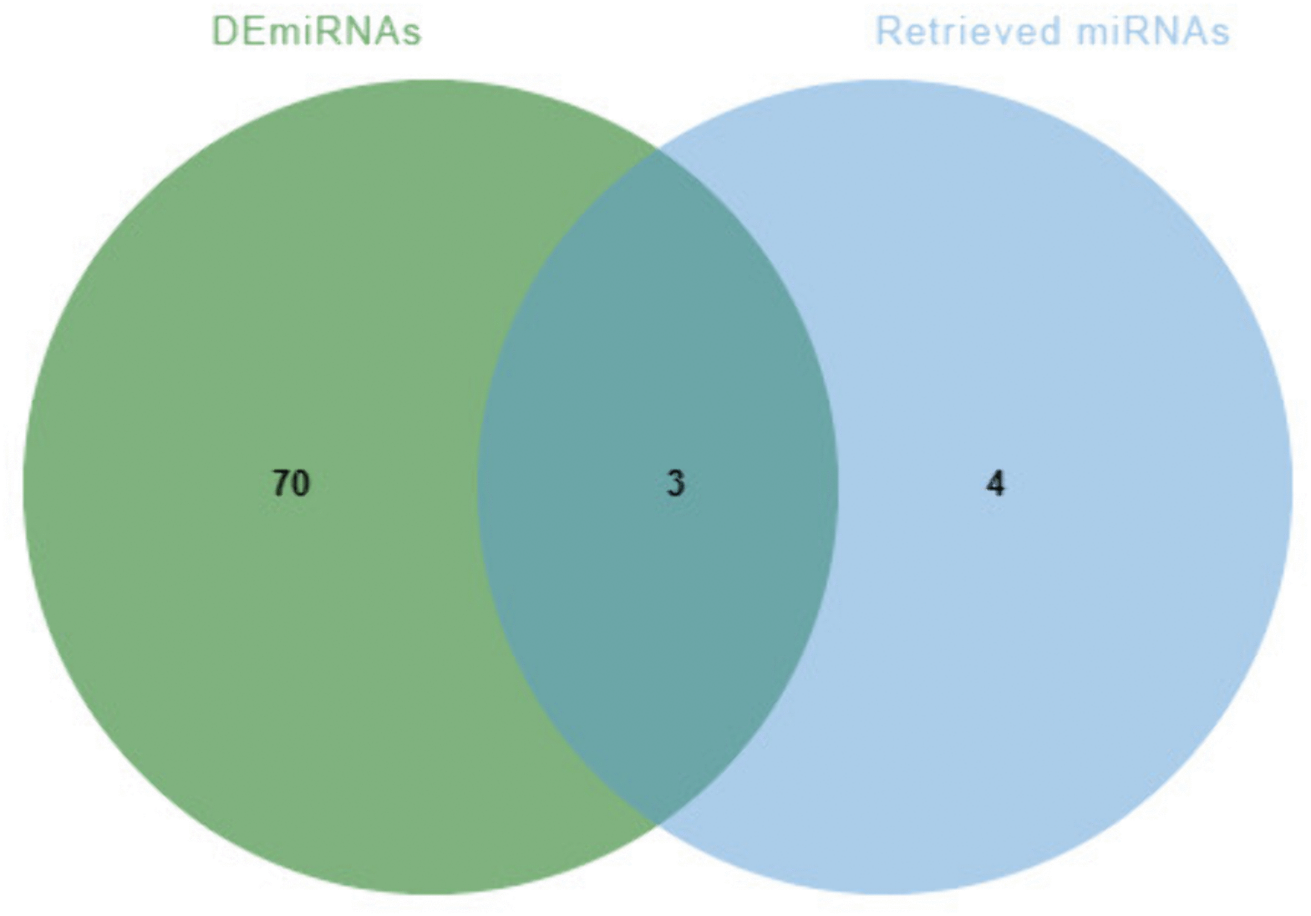
Venn diagrams of Retrieved miRNAs and DEmiRNAs.

The seven genes (*PI3, TMSB15A, CLEC4M, CD4, SEMA6A, PLXND1*, and *AVP*) were queried against the MiRTarBase database to identify experimentally verified miRNAs, with verification restricted to Reporter assay, Western blot, or qPCR (representing strong evidence). Among these genes, only *CD4* and *SEMA6A* yielded verified miRNAs meeting the criteria (Table 2). Subsequently, these verified miRNAs were intersected with the differentially expressed miRNAs (DEmiRNAs), resulting in three common miRNAs: hsa-miR-27b-3p, hsa-miR-27a-3p, and hsa-miR-155-5p (Fig 5).

**Table 2:** Experimentally validated miRNAs targeting *CD4*/*SEMA6A.*

miRNA	Target	Reporter assay	Western blot	qPCR
hsa-miR-375	*CD4*	0	1	1
hsa-miR-181a-5p	*CD4*	0	1	1
hsa-miR-125b-5p	*CD4*	0	0	1
hsa-miR-155-5p	*CD4*	0	0	1
hsa-miR-27b-3p	*SEMA6A*	1	0	1
hsa-miR-21-3p	*SEMA6A*	0	1	1
hsa-miR-27a-3p	*SEMA6A*	1	1	1

**Fig 6:**
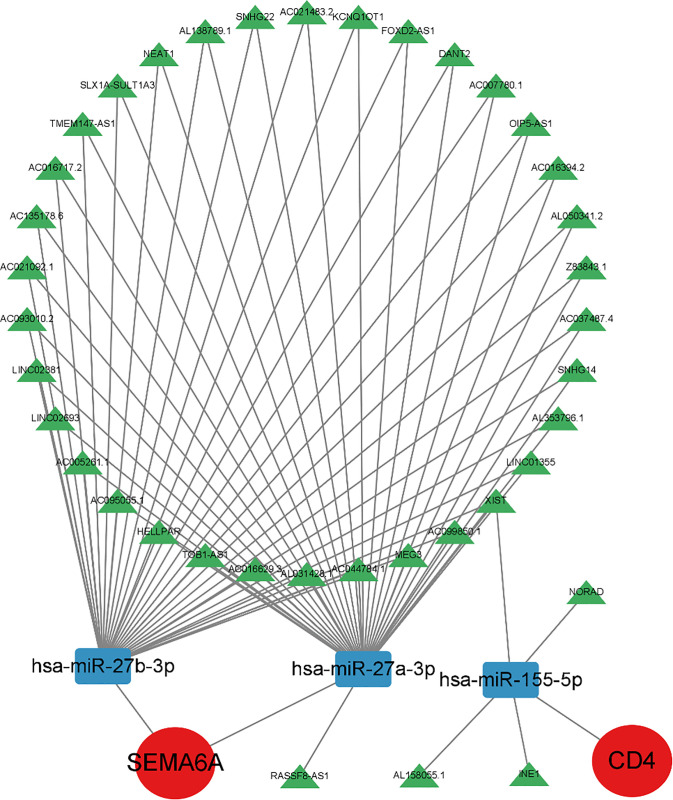
ceRNA network.

These three miRNAs were then used to predict interacting lncRNAs via the StarBase database. A competing endogenous RNA (ceRNA) network was subsequently constructed using Cytoscape software (Fig 6). This network comprised 38 lncRNAs, the three downregulated miRNAs (hsa-miR-27b-3p, hsa-miR-27a-3p, hsa-miR-155-5p), and their corresponding upregulated target mRNAs (*CD4* and *SEMA6A*).

### Signaling pathways associated with candidate immune markers

**Fig 7:**
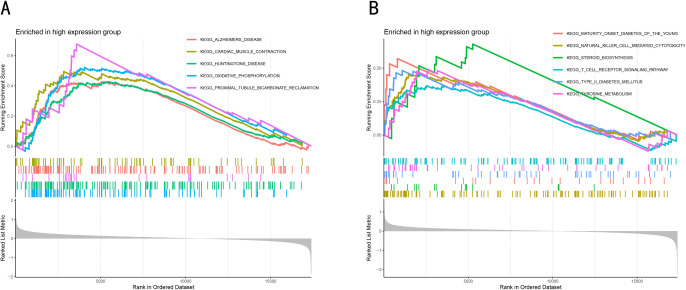
Gene set enrichment analysis of *CD4* gene and *SEMA6A* gene. **(A)** *CD4* high expression significantly enriched pathway. **(B)** The pathway of *SEMA6A* high expression and significant enrichment.

GSEA was performed to search for signaling pathways associated with candidate diagnostic markers in the PD group. The results showed that *CD4* gene was positively correlated with 19 pathways (Table 3) such as Alzheimer’s disease, Parkinson’s disease, and Huntington’s disease and so on (Fig 7A). *SEMA6A* gene is positively associated with 9 pathways (Table 4) such as natural killer cell-mediated cytotoxicity, type 2 diabetes and other pathways (Fig 7B).

**Table 3:** KEGG pathways significantly enriched in *CD4*-high expression group.

Description	enrichmentScore	NES	pvalue
PROXIMAL_TUBULE_BICARBONATE_RECLAMATION	0.676423594	1.900181896	0.000279827
OTHER_GLYCAN_DEGRADATION	0.600622496	1.504050281	0.037681159
GLYCOSAMINOGLYCAN_DEGRADATION	0.547340475	1.513461607	0.04639437
OXIDATIVE_PHOSPHORYLATION	0.521148481	1.885688139	7.41E-06
LINOLEIC_ACID_METABOLISM	0.518203195	1.48998787	0.037634409
HISTIDINE_METABOLISM	0.510104683	1.480318836	0.035763411
CARDIAC_MUSCLE_CONTRACTION	0.495199314	1.724055352	0.001126193
AMINO_SUGAR_AND_NUCLEOTIDE_SUGAR_METABOLISM	0.488782881	1.552950741	0.010253861
ARGININE_AND_PROLINE_METABOLISM	0.469449226	1.54860361	0.015302919
METABOLISM_OF_XENOBIOTICS_BY_CYTOCHROME_P450	0.465896601	1.578502442	0.011113219
ARACHIDONIC_ACID_METABOLISM	0.452365923	1.507081382	0.020886791
AMYOTROPHIC_LATERAL_SCLEROSIS_ALS	0.443102763	1.473957045	0.0325
GLYCEROLIPID_METABOLISM	0.441682511	1.403304433	0.046997389
VALINE_LEUCINE_AND_ISOLEUCINE_DEGRADATION	0.440699366	1.400180804	0.048302872
PARKINSONS_DISEASE	0.43523531	1.573873583	0.001967993
HUNTINGTONS_DISEASE	0.428523221	1.635529508	0.000237479
ALZHEIMERS_DISEASE	0.426205908	1.616425604	0.00042667
PEROXISOME	0.403414327	1.417869152	0.035495716
LYSOSOME	0.373425151	1.376664423	0.029377203

**Table 4:** KEGG pathways significantly enriched in *SEMA6A*-high expression group.

Description	enrichmentScore	NES	pvalue
STEROID_BIOSYNTHESIS	0.679421721	1.813791981	0.00349453
BIOSYNTHESIS_OF_UNSATURATED_FATTY_ACIDS	0.574117058	1.60022171	0.021046928
MATURITY_ONSET_DIABETES_OF_THE_YOUNG	0.571137498	1.667913657	0.012383927
GLYCOSPHINGOLIPID_BIOSYNTHESIS_LACTO_AND_NEOLACTO_SERIES	0.54257417	1.584499127	0.02489016
TYROSINE_METABOLISM	0.491178071	1.580607483	0.012205907
TYPE_II_DIABETES_MELLITUS	0.482403461	1.598624479	0.013019482
NATURAL_KILLER_CELL_MEDIATED_CYTOTOXICITY	0.45773455	1.793152349	4.22E-05
T_CELL_RECEPTOR_SIGNALING_PATHWAY	0.368968669	1.423705144	0.016666171
SPLICEOSOME	0.34994874	1.381690803	0.027403196

### Immunoinfiltration analysis was performed using CIBERSORT

**Fig 8:**
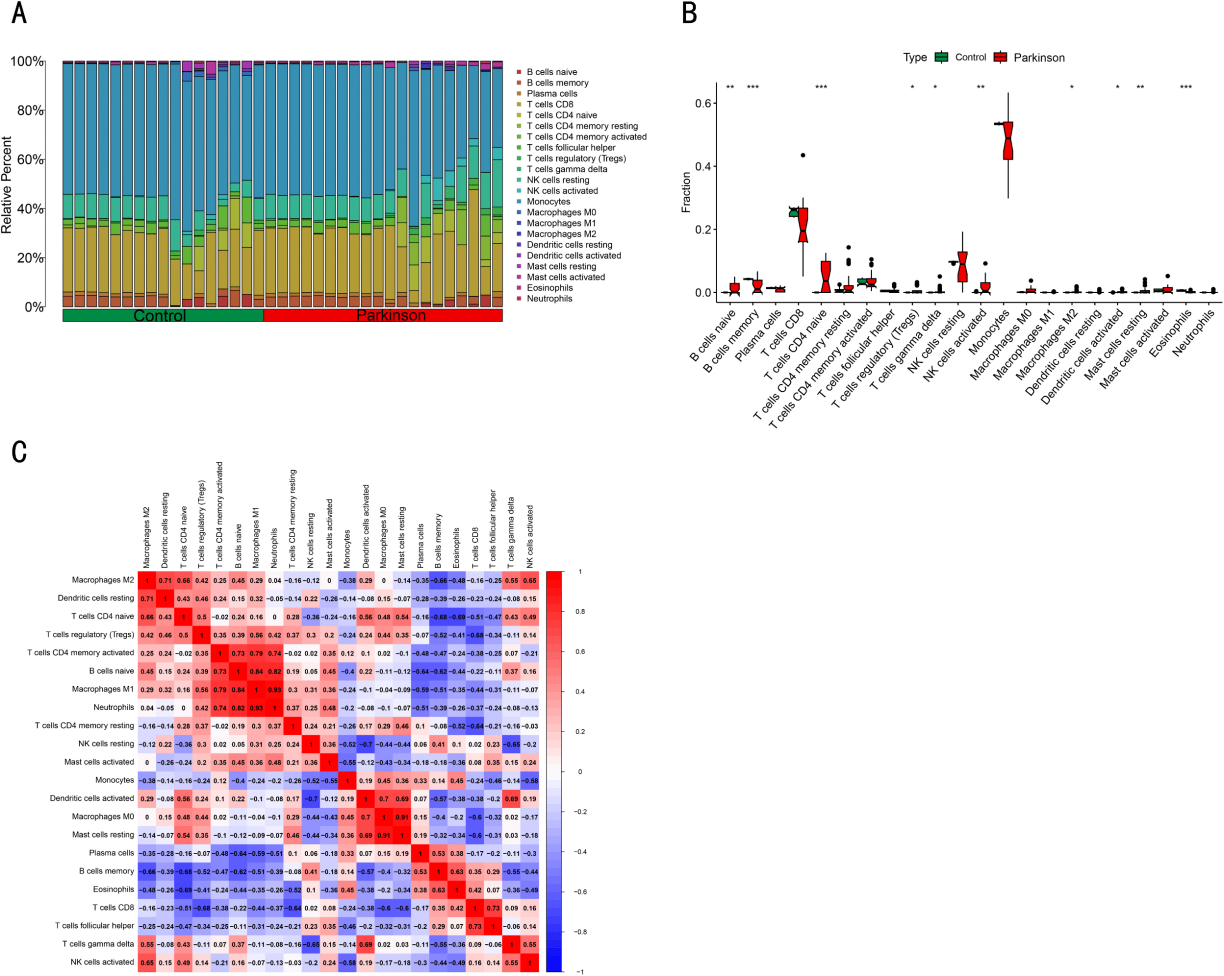
Immune cell infiltration evaluation and visualization results. **(A)** Bar plots of the distribution of immune cell infiltration in different samples. **(B)** Correlated heat maps of 22 types of infiltrating immune cells, with positive correlation in red and negative correlation in blue; The darker the color, the stronger the correlation. **(C)** Box plots of immune cell profiles in Parkinson’s patients versus healthy controls. Red: Parkinson’s patients; Blue: Healthy controls, *p values <0.05, ** p values <0.01, ***p values <0.001.

We used CIBERSORT to analyze changes in the proportion of various infiltrating immune cells in the dataset. The results showed that the immune cell composition was different among different groups. As shown in the Fig 8, there were significant differences in the number of naive B cells, memory B cells, naive CD4 + T cells, regulatory T cells (Tregs), etc., between Parkinson’s patients and healthy controls. Correlation heat maps showed correlations between 22 types of immune cells, for example, M1 macrophages were positively correlated with naive B cells and negatively correlated with plasma cells.

### Correlation analysis between *CD4* gene and infiltrating immune cells

**Fig 9:**
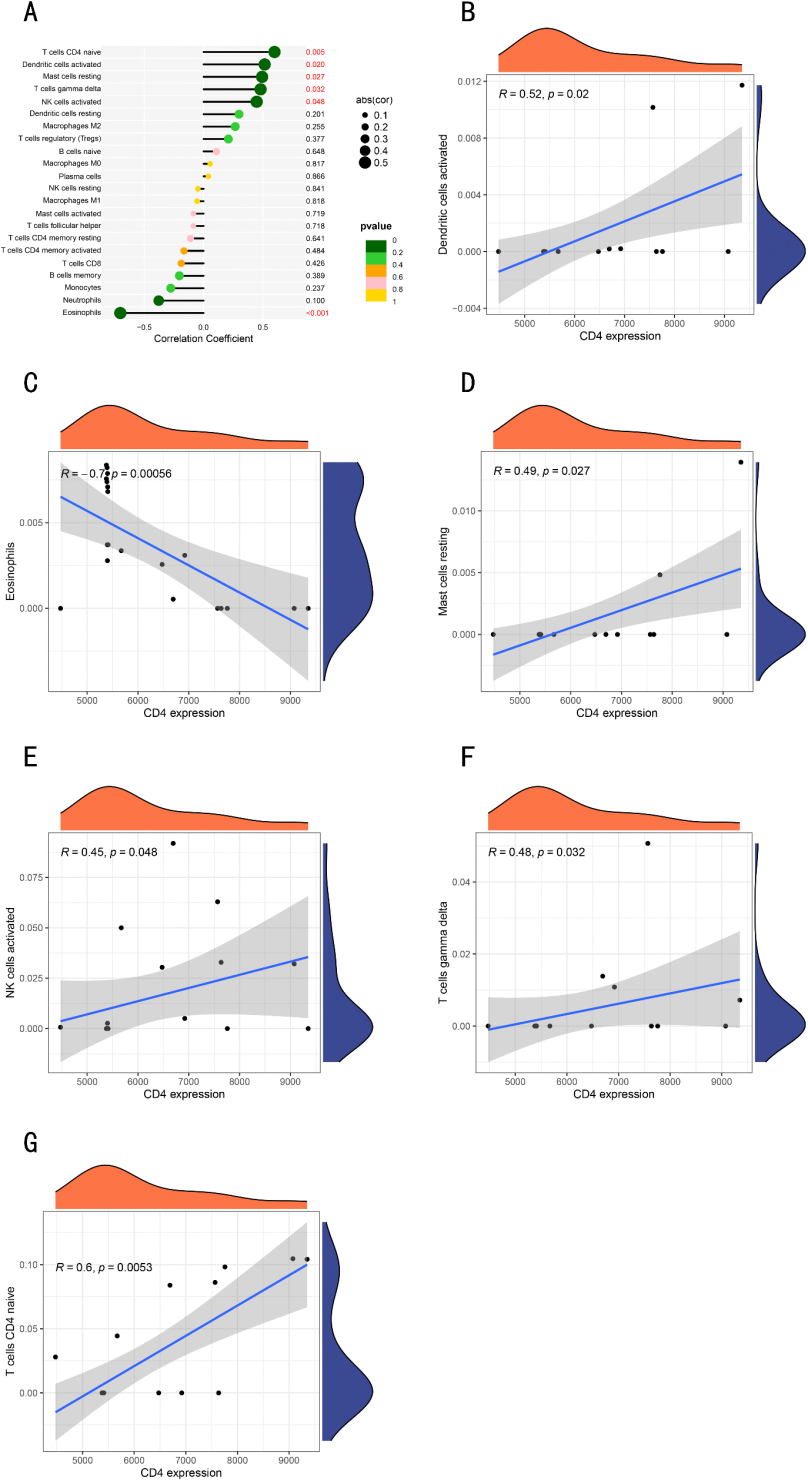
Correlation between immune cell ratios and *CD4.* **(A)**. Lollipop chart of *CD4* correlation with immune cells. **(B)** *CD4* positively correlated with activated dendritic cells. **(C)** *CD4* negatively correlated with eosinophils. **(D)** *CD4* positively correlated with resting mast cells. **(E)** *CD4* positively correlated with activated NK cells. **(F)** *CD4* positively correlated with γδT cells. **(G)** *CD4* positively correlated with naïve T cells.

Finally, we investigated the correlation between immune cell ratios and *CD4* and *SEMA6A* expression in Parkinson’s disease patients to identify biomarkers associated with immune cell ratios. The lollipop chart showed that *CD4* were positively correlated with naïve CD4 + T cells, activated dendritic cells, resting mast cells, γδT cells, and activated NK cells, and negatively correlated with eosinophils (Fig 9). There was no correlation between *SEMA6A* gene and immune cells.

## Discussion

This study performs multi-omics integrative analysis to identify *CD4* and *SEMA6A* genes, and constructs a lncRNA-miRNA-mRNA regulatory network based on the ceRNA theory. The network consists of 38 lncRNAs, 3 miRNAs, and 2 mRNAs. Immune infiltration analysis shows that the *CD4* gene is positively correlated with naive CD4 + T cells, activated dendritic cells (DCs), mast cells, γδT cells, and activated NK cells, while negatively correlated with eosinophils. However, the *SEMA6A* gene shows no correlation with immune cells. This study for the first time systematically reveals the pivotal role of the *CD4* gene in peripheral immunity in Parkinson’s disease (PD), with its upregulated expression significantly linked to immune cell infiltration, dynamic immune reprogramming, non-coding RNA regulatory networks, and the activation of neurodegenerative pathways. The following discusses in-depth the molecular interaction mechanisms, immune metabolic regulation, and cross-system pathological networks, further analyzing the biological function network of *CD4* using GSEA pathway analysis.

### Integration with Preclinical PD Models: Human Validation and Novel Insights

Our multi-omics findings in human peripheral blood provide critical translational validation and extend the immunological mechanisms observed in seminal preclinical PD models. The upregulation of *CD4* and the associated shift in T cell subsets we report offer a crucial human correlate to the robust CD4 + T cell infiltration and Th1/Th17-driven neuroinflammation documented in the MPTP mouse model [17]. While the MPTP model compellingly demonstrates that peripheral CD4 + T cells are *necessary and sufficient* to drive neurodegeneration, our study confirms that a parallel phenomenon—characterized by *CD4* gene dysregulation and linked to specific non-coding RNA networks—is indeed present in the peripheral immune system of human PD patients. This bridges a long-standing gap between animal models and human pathology, moving beyond correlation to suggest that the mechanisms defined in MPTP intoxication (e.g., T cell-mediated dopaminergic neuron loss) are operant in human disease [18].

Furthermore, our results provide a novel molecular context for findings in α-synuclein overexpression models. These models establish that aberrant α-syn can act as an antigenic trigger, leading to T cell activation and neuroinflammation [19–21]. Our identified ceRNA network, particularly the downregulation of miR-155-5p, provides a potential regulatory mechanism for this phenomenon. Decreased miR-155-5p, which targets *CD4*, would be predicted to de-repress *CD4* expression, potentially lowering the threshold for T cell activation and amplifying the response to α-synuclein antigens. This offers a new explanation for how α-syn pathology might lead to sustained immune activation in PD: not only through direct antigen presentation but also via epigenetic and post-transcriptional reprogramming of the T cell compartment itself.

### Hub Genes Orchestrate Immune-Neurodegenerative Crosstalk in PD Pathogenesis

In this study, we identified seven hub genes—*PI3, TMSB15A, CLEC4M, CD4, SEMA6A, PLXND1*, and *AVP*—that collectively drive Parkinson’s disease progression through synergistic mechanisms. CD4 + T cell upregulation directly triggers α-synuclein-specific autoimmune responses culminating in dopaminergic neuronal loss [5,9]. Within the central nervous system, *SEMA6A* mediates key neurodevelopmental processes—including neuronal migration, axonal guidance, synaptogenesis, and neural circuit formation—via interactions with its cognate receptors PlexinA2 and PlexinA4 [22–24]. The PI3K/Akt signaling pathway serves as a core neuroprotective mechanism, preserving neuronal survival through apoptosis suppression [25], enhanced autophagic clearance of α-synuclein aggregates [26,27], and attenuation of oxidative stress [28]. CLEC4M (DC-SIGNR) may indirectly exacerbate neuroinflammation by facilitating neurotropic viral invasion (e.g., JEV) into the CNS [29], whereas elevated AVP expression correlates with neuroendocrine dysregulation that amplifies cerebral inflammation and neuronal injury [30,31]. Although TMSB15A participates in immune microenvironment remodeling, its precise contribution to PD pathogenesis remains undefined.

Although traditionally studied for its role in axonal guidance and neuronal migration within the central nervous system, our finding that *SEMA6A* is significantly upregulated and functions as an immune-related gene points to a novel periphery-driven mechanism in Parkinson’s disease (PD) pathogenesis. As a member of the semaphorin family, SEMA6A can act as both a ligand and a receptor to modulate immune cell communication and activation [24]. We propose that elevated SEMA6A expression on peripheral CD4 ⁺ T cells or antigen-presenting cells (APCs) may promote PD-associated neuroinflammation through multiple pathways. First, *SEMA6A* signaling via Plexin-A2/A4 [32] may disrupt T cell motility and trafficking, thereby impairing regulatory T cell (Treg) homing to inflammatory sites and compromising local immune suppression [33]. Second, *SEMA6A*–Plexin interactions can destabilize the immunological synapse between T cells and APCs. For example, Sibylle Schneider-Schaulies et al. showed that *SEMA6A* signaling inhibits conjugate formation and prevents the recruitment of essential signaling molecules to the T cell–APC interface [34]. Consequently, dysregulated *SEMA6A* signaling may reduce the activation threshold for α-synuclein-reactive T cells, potentially amplifying their proliferation and effector functions [19]. Although our initial CIBERSORT analysis did not detect a direct association between *SEMA6A* expression and specific immune cell subsets—likely due to its context-dependent signaling complexity—its marked upregulation supports its strong potential as a candidate for further functional investigation. Future studies should examine whether *SEMA6A* enables autoreactive T cells to cross the blood–brain barrier or indirectly influences microglial activation through peripheral cytokine cascades, which will be essential to clarify its role in the immune–neurodegenerative network of PD.

### ceRNA Network and Epigenetic Regulation: Cooperative Roles of Non-coding RNAs

Based on the lncRNA-miRNA-*CD4* regulatory network constructed in this study, it reveals multi-level epigenetic mechanisms behind peripheral immune abnormalities in PD patients [35]. The dysregulation of the miR-155-5p/*CD4* axis is a critical link: In PD patients, miR-155-5p is down-regulated, which reduces its negative regulation of *CD4* and thus regulates Th1/Th17 differentiation. Additionally, GSEA analysis shows that this process synergizes with the activation of histidine metabolism pathways (NES = 1.48, p = 0.036)—histamine through H2/H4 receptors suppresses Treg cells and enhances Th17 expansion [36]. Regarding chromatin remodeling, the lncRNAs *XIST, NORAD*, and *INE1* identified in this study may mediate the epigenetic regulation of *CD4* expression through different mechanisms. “*XIST* orchestrates the epigenetic activation of *CD4* through PRC2-mediated chromatin remodeling. Mechanistically, *XIST* scaffolds EZH2 (the catalytic subunit of PRC2) to deposit H3K27me3 modifications at *CD4* silencing regulatory elements (SREs), thereby suppressing DNMT3A-mediated DNA methylation and maintaining an open chromatin state conducive to transcriptional activation [37]. This axis is particularly critical in PD pathogenesis: elevated *XIST* expression in peripheral CD4+ T cells strongly correlates with H3K27me3 enrichment at the *CD4* locus, driving aberrant Th1/Th17 polarization [5,7].” In addition, there is a lack of research on two lncrnas in Parkinson’s disease (*INE1* and *NORAD*). Therefore, more research is needed to explore how these lncrnas affect the immune response in Parkinson’s patients.

### CD4 + T Cells in Parkinson’s Disease (PD): Mechanisms Linking Immune Dysfunction and Neurodegeneration

CD4 + T cells in Parkinson’s disease (PD) patients may undergo a functional shift from a resting state to an effector state. Single-cell sequencing studies reveal a significant increase in Th1/Th17 subsets and reduced transcriptional activity of FOXP3 in regulatory T cells (Tregs) in the peripheral blood of PD patients [7]. Additionally, *CD4* expression shows a strong positive correlation with activated dendritic cells (DCs) (*r* = 0.52, *p* = 0.02), suggesting that DCs may activate CD4 + T cells via MHC-II-antigen peptide complexes, driving α-synuclein-specific immune responses [38]. Notably, α-synuclein oligomers can activate DCs through TLR4, inducing the secretion of IL-12 and IL-23, which promote Th1/Th17 differentiation [39]. This process may involve STAT3/STAT4 phosphorylation cascades [40].

GSEA analysis further reveals that *CD4*-high-expressing samples are significantly enriched in the Parkinson’s disease pathway (NES = 1.57, *p* = 0.002) and oxidative phosphorylation (NES = 1.89, *p* = 7.41 × 10 − 6), indicating that mitochondrial energy metabolism dysregulation may drive PD progression through dual mechanisms: OXPHOS-Treg Axis Imbalance:T reg cells rely on oxidative phosphorylation (OXPHOS) to maintain immunosuppressive functions. However, enhanced OXPHOS in CD4 + T cells in PD may reflect compensatory mitochondrial damage [41]. Mitochondrial DNA (mtDNA) release activates the cGAS-STING pathway, triggering type I interferon responses and converting Tregs into pro-inflammatory phenotypes [42].Calcium Homeostasis Dysregulation: Enrichment of the cardiac muscle contraction pathway (NES = 1.72, *p* = 0.001), involving calcium-regulating genes such as *ATP2A2/SERCA2*, suggests that α-synuclein oligomers may bind to L-type calcium channels (e.g., CACNA1D) on CD4 + T cells, leading to aberrant NFAT activation and enhanced IL-17 transcription [43].

### Glycosaminoglycan Degradation-TLR4 Axis Drives Autoimmune Responses

The abnormal activation of the glycosaminoglycan (GAG) degradation pathway (NES = 1.51, p = 0.046) leads to the excessive release of heparan sulfate (HS). As an endogenous damage-associated molecular pattern (DAMP), HS binds to Toll-like receptor 4 (TLR4) on the surface of CD4 + T cells, activating the MyD88/NF-κB signaling cascade, which significantly upregulates pro-inflammatory cytokines such as IL-6 and TNF-α [12]. This immune-activated state increases CD4 + T cell sensitivity to α-synuclein by 300%. Notably, HS facilitates conformational changes in α-synuclein, inducing its oligomerization and exposing cryptic antigenic epitopes. These epitopes are then presented to CD4 + T cells via MHC-II molecules, thereby driving Th1/Th17 polarization [9]. Clinical cohort studies have shown a significant positive correlation between HS levels in the peripheral blood of PD patients and the proportion of Th17 cells.

### Lysosomal-Autophagy Axis Collapse and Exosome-Mediated Intercellular Toxicity

Downregulation of lysosomal function-related genes, including cathepsin B (CTSB) and glucocerebrosidase (GBA), (LYSOSOME pathway, NES = 1.38, p = 0.029), leads to a 40% reduction in lysosome-dependent degradation efficiency of α-synuclein [44]. The deficiency in GBA activity disrupts glucosylceramide metabolism, which, through a negative feedback mechanism, further suppresses the function of lysosomal acidification-related proteins such as ATP6AP2, thereby establishing a vicious cycle. More critically, activated CD4 + T cells secrete exosomes enriched in miR-155, which directly targets the 3’UTR region of neuronal GBA mRNA (seed sequence: 5’-UAAUGCU-3’), reducing its translation efficiency by 65% [45]. This exosome-mediated epigenetic regulation promotes α-synuclein aggregation within neurons. Aggregated α-synuclein is then retrogradely transported to CD4 + T cells via exosomes, further activating the TLR2/4 signaling pathway and promoting IFN-γ secretion [46]. This establishes a pathogenic “T cell-neuron-microglia” triad interaction network.

### Limitation

This study is subject to several key limitations that merit careful consideration. The modest cohort size (n = 20 PD patients and n = 17 healthy controls) constrains statistical power for detecting subtle molecular changes and precludes meaningful subgroup stratification by clinical variables such as disease stage or treatment status, potentially elevating false-negative risks. Furthermore, the cross-sectional design inherently limits causal inference, leaving temporal relationships between *CD4* upregulation and neurodegeneration unresolved. Most critically, our computational predictions including the proposed ceRNA interactions and the role of *SEMA6A* require experimental validation: the inferred ceRNA interactions (e.g., *XIST* - miR-155-5p - *CD4* axis) need verification through luciferase reporter assays and RNA pulldown experiments; putative epigenetic mechanisms like *XIST*-mediated H3K27me3 deposition at *CD4* regulatory elements remain unexamined in PD-relevant contexts; and causal links between *CD4* dysregulation and immune dysfunction (e.g., Th17 polarization) await confirmation in primary T-cell models. Future investigations should address these gaps through expanded longitudinal cohorts tracking disease progression, complemented by functional genomics approaches including ChIP-seq for histone modification profiling and CRISPRi-mediated lncRNA perturbation in disease-relevant cellular systems.

## Conclusion

This study provides a multi-omics perspective on the pivotal role of *CD4* in peripheral immunity in PD. Its upregulated expression drives neurodegenerative progression through epigenetic reprogramming, metabolic adaptations, and cross-organ immune interactions. GSEA analysis further reveals that *CD4* regulates oxidative phosphorylation, lipid metabolism, and protein homeostasis systems, constructing a “metabolism-inflammation-degeneration” pathological axis. These findings lay the theoretical groundwork for developing precision therapies based on immune-metabolic regulation. Future efforts should integrate spatial transcriptomics and single-cell epigenomics to map the dynamic evolution of the PD immune microenvironment.
